# Supplementary material for: Novel Driver Strength Index highlights important cancer genes in TCGA PanCanAtlas patients
Source: PeerJ. 2022 Aug 11;10:e13860. doi: 10.7717/peerj.13860 (PMC9375969; doi:10.7717/peerj.13860)
Supplement: Supplemental Information 2 [file peerj-10-13860-s002.zip › COHORTS/genes plots/cohorts/2021_8_16_14_9_distribution_cohorts_females_THYM.pdf]

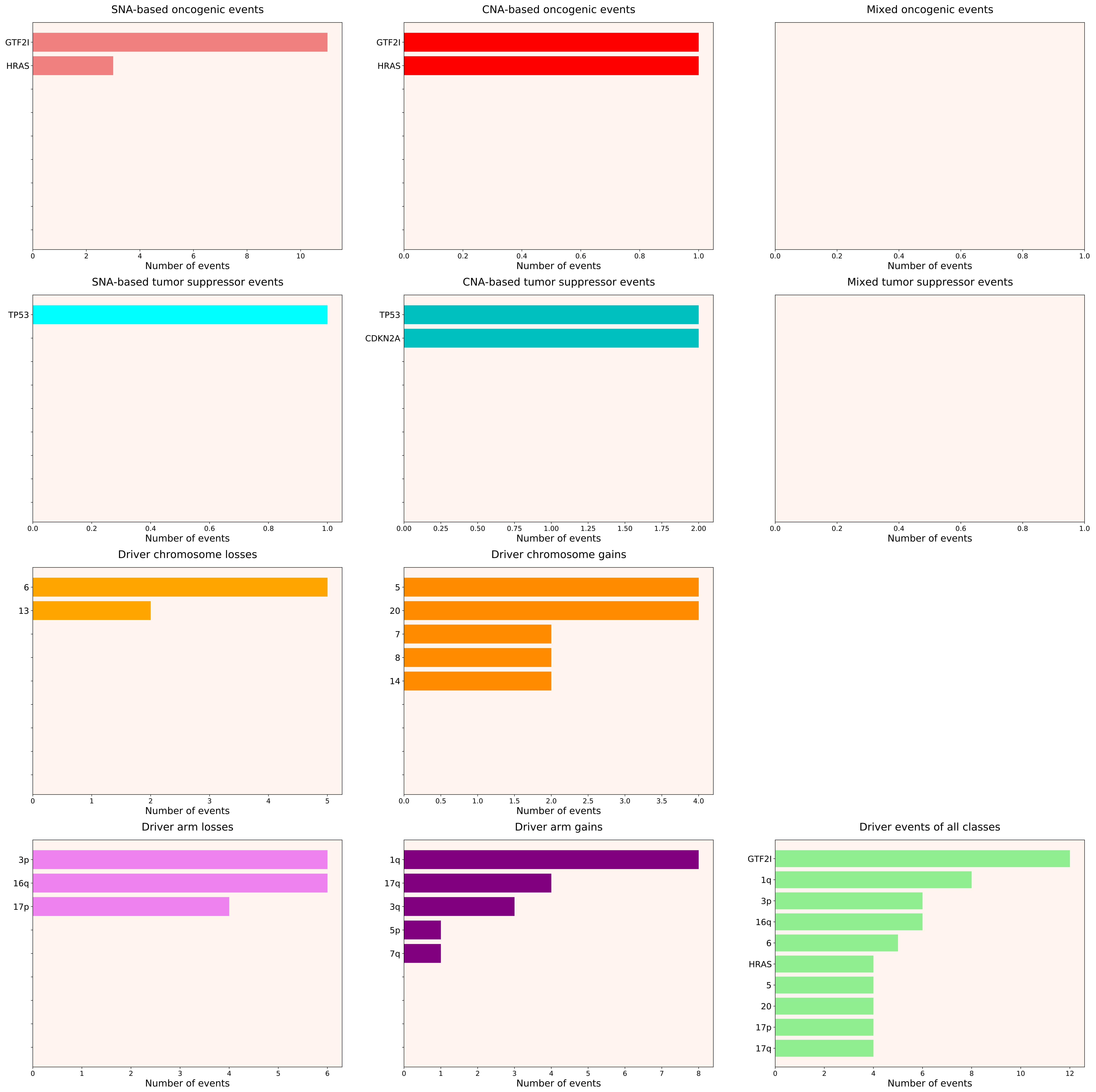
SNA-based oncogenic events
GTF2I
HRAS
Number of events
CNA-based oncogenic events
GTF2I
HRAS
Number of events
Mixed oncogenic events
Number of events
SNA-based tumor suppressor events
TP53
Number of events
CNA-based tumor suppressor events
TP53
CDKN2A
Number of events
Mixed tumor suppressor events
Number of events
Driver chromosome losses
6
13
Number of events
Driver chromosome gains
5
20
7
8
14
Number of events
Driver arm losses
3p
16q
17p
Number of events
Driver arm gains
1q
17q
3q
5p
7q
Number of events
Driver events of all classes
GTF2I
1q
3p
16q
6
HRAS
5
20
17p
17q
Number of events
